# Comparative Performance of the Luminex NxTAG Respiratory Pathogen Panel, GenMark eSensor Respiratory Viral Panel, and BioFire FilmArray Respiratory Panel

**DOI:** 10.1128/spectrum.01248-22

**Published:** 2022-06-29

**Authors:** Elena B. Popowitch, Sam Kaplan, Zenglin Wu, Yi-Wei Tang, Melissa B. Miller

**Affiliations:** a Clinical Microbiology Laboratory, McLendon Clinical Laboratories, UNC Health Care, Chapel Hill, North Carolina, USA; b Department of Laboratory Medicine, Memorial Sloan Kettering Cancer Center, New York, New York, USA; c Department of Laboratory Medicine, The Eighth Affiliated Hospital of Sun Yat-sen University, Shenzhen, China; d Department of Pathology and Laboratory Medicine, University of North Carolina School of Medicine, Chapel Hill, North Carolina, USA; Johns Hopkins Hospital

**Keywords:** respiratory viral panels, respiratory pathogen panels, BioFire, GenMark, Luminex, syndromic testing

## Abstract

This study compares three of the most inclusive and widely used panels for respiratory syndromic testing in the United States, namely, Luminex NxTAG Respiratory Pathogen Panel (RPP), BioFire FilmArray Respiratory Panel (RP), and GenMark eSensor Respiratory Viral Panel (RVP). We compared the three assays using nasopharyngeal swab samples (*n* = 350) collected from symptomatic patients (*n* = 329) in the pre-coronavirus disease 2019 (COVID-19) era. There was no significant difference in the overall accuracies of BioFire and Luminex assays (*P* = 0.6171); however, significant differences were found between BioFire and GenMark (*P* = 0.0003) and between GenMark and Luminex (*P* = 0.0009). The positive percent agreement of the BioFire RP assay was 94.1%, compared to 97.3% for GenMark RVP and 96.5% for Luminex RPP. Overall negative percent agreement values were high for all three assays, i.e., 99.9% for BioFire and Luminex and 99.5% for GenMark. The three assays were equivalent for adenovirus, human metapneumovirus, influenza A, and respiratory syncytial virus. Increased false-positive results were seen with BioFire for the endemic coronaviruses and with GenMark for influenza B and the parainfluenza viruses.

**IMPORTANCE** Clinical laboratories have multiple choices when it is comes to syndromic respiratory testing. Here, the Luminex NxTAG RPP is compared to the BioFire FilmArray RP and GenMark eSensor RVP for overall and per-target accuracy. As new tests come to market, it is important to ascertain their performance characteristics, compared to other widely used *in vitro* diagnostic products.

## INTRODUCTION

Acute respiratory infections continue to be major drivers of health care visits in the United States. While overall mortality rates due to influenza and pneumonia have been decreasing (1), the burden of respiratory illness in the United States remains significant and will remain so as mitigation strategies, such as masking, decline from initial coronavirus disease 2019 (COVID-19) pandemic efforts. In the pre-COVID-19 era, viral infections were detected in 69% of adults and 90% of children hospitalized for community-associated pneumonia (2, 3). Clinical differentiation of acute respiratory infections is difficult, because the overlap of signs and symptoms is significant. Accurate identification of the causative agent is important, because acute respiratory infections are the leading complaint for which antibiotics are prescribed for adults (4). Diagnosis beyond influenza and respiratory syncytial virus (RSV) may predict the severity of the illness (5) and inform proper cohorting of patients (6). Syndromic multiplexed molecular panels offer detection and identification of many of the most common viral (and in some cases bacterial) pathogens from a single patient sample. The appeal to both the clinician and the laboratorian is understandable, as testing becomes more streamlined and results more comprehensive.

The three most inclusive respiratory pathogen panels (RPPs) cleared by the FDA at the time of this study are the FilmArray Respiratory Panel (RP) (BioFire Diagnostics, Salt Lake City, UT), the eSensor Respiratory Viral Panel (RVP) (GenMark Diagnostics, Carlsbad, CA), and the NxTAG RPP (Luminex Molecular Diagnostics, Austin, TX) (7). Separate studies comparing one assay to another or to a reference method have generally found high levels of sensitivity and specificity (8). To date, the Luminex NxTAG RPP has been compared to Luminex xTAG RVP (9), Luminex xTAG RVP FAST v2 (10–12), BioFire RP (7, 13), RespiFinder-22 (14), Anyplex II RV16 (15), and laboratory-developed tests (LDTs) (12, 13, 16). However, there has not been a comparison of the NxTAG RPP to two widely used RPs in the United States, namely, BioFire RP and GenMark eSensor RVP. We compared all three assays using the same set of nasopharyngeal (NP) swab samples collected from symptomatic patients. Notably, the versions of the tests compared do not include severe acute respiratory syndrome coronavirus 2 (SARS-CoV-2), which allows us to focus on non-COVID-19 respiratory pathogen performance.

## RESULTS

Of the 350 NP swab samples tested, 199 were consensus positive for a single viral target, 24 were positive for two viral targets, and 3 were positive for three viral targets (Table 1). No samples were positive for more than three targets. There were 139 consensus negative results, 100 of which were negative by all three tests. Within the consensus positive results were a combined 31 false-negative results (i.e., results that were positive by two assays and negative by the third). The false-negative results were split between the assays; Luminex RPP had 15 false-negative results, GenMark RVP had 7, and BioFire RP had 9 (Table 1). The distribution of false-positive results, or those analytes detected by only one of the three assays, was skewed more to the GenMark RVP with 27 false-positive results, while the BioFire and Luminex assays tallied 7 and 5 false-positive results, respectively (Table 2).

**TABLE 1:** Results of testing according to analyte (*n* = 256 consensus positive targets)

Target	No. of samples with BF/GM/LX^*a*^ results of:			
	+/+/+	+/+/−	+/−/+	−/+/+
Adenovirus	12	1		2
CoV 229E	4			
CoV HKU1	14	2		
CoV NL63	10		1	
CoV OC43	11			
MPV	22			
Influenza A 2009-H1N1	21^*b*^			
Influenza A H3	9			1
Influenza B	14			1
PIV-1	8			1
PIV-2	3			
PIV-3	10			
PIV-4	5	2		2
Rhinovirus/enterovirus	50	4	6	6
RSV A	22			2
RSV B	10			
Total	225	9	7	15

a BF, BioFire FilmArray RP; GM, GenMark eSensor RVP; LX, Luminex NxTAG RPP.

b Two samples were influenza A positive, subtype negative, by BioFire.

**TABLE 2:** Breakdown of false-positive results (*n* = 39)

Target	No. of samples with BF/GM/LX^*a*^ results of:		
	+/−/−	−/+/−	−/−/+
Adenovirus		1	
CoV 229E	1		
CoV HKU1	1		
CoV NL63			
CoV OC43	4	1	
MPV	1	1	2
Influenza A			1 (not typed)
Influenza B		4	
PIV-1		2	
PIV-2		3	
PIV-3			
PIV-4		4	
Human rhinovirus/enterovirus		10	1
RSV A		1	
RSV B			1
Total	7	27	5

a BF, BioFire FilmArray RP; GM, GenMark eSensor RVP; LX, Luminex NxTAG RPP.

### Positive and negative percent agreements.

Overall positive and negative agreements were calculated for each assay. To compare the assays fairly, the metrics were assessed for the 16 targets they have in common, omitting bocavirus and the bacterial pathogens. The positive percent agreement (PPA) of the BioFire RP assay was 94.1%, compared to 97.3% for GenMark RVP and 96.5% for Luminex RPP. Chi-square analysis found no dissimilarity in these values (*P* = 0.9728). Overall negative percent agreement (NPA) was high for all three assays, i.e., 99.9% for BioFire and Luminex and 99.5% for GenMark. To determine the overall performance, the total number of possible results was established by multiplying the number of targets detectable (BioFire, 19 targets; GenMark, 18 targets; Luminex, 21 targets) by the number of NP swab samples tested (*n* = 350). PPA was 94.2% for BioFire, 96.3% for Luminex, and 97.3% for GenMark. NPA was consistent at 99.6% (GenMark) and 99.9% (BioFire and Luminex).

### Accuracy.

McNemar’s tests were performed to evaluate whether significant differences existed between any two of the assays. There was no significant difference in accuracy between BioFire and Luminex (*P* = 0.6171); however, significant differences were found between BioFire and GenMark (*P* = 0.0003) and between GenMark and Luminex (*P* = 0.0009). To investigate the source of the differences, assay disparities for each analyte or analyte group were analyzed (see Table S1 in the supplemental material). The three assays were equivalent for adenovirus, human metapneumovirus (MPV), influenza A, and RSV. BioFire had increased false-positive results for the endemic coronaviruses (CoVs) and so was found to be less accurate for that target group. The same was true for GenMark with influenza B and the parainfluenza viruses (PIVs). Rhinovirus/enterovirus presented an analytical challenge. The GenMark RVP assay reportedly does not detect enteroviruses; therefore, the 6 true-positive specimens for rhinovirus/enterovirus not detected by GenMark might have been enteroviruses and not false-negative results for rhinovirus.

### Adenovirus.

In addition to the BioFire, GenMark, and Luminex assays, each sample was separately extracted and tested for adenovirus using an LDT. All 15 consensus positive specimens were LDT positive for adenovirus; the specimen found to be adenovirus positive by GenMark alone was negative. No other specimens tested positive for adenovirus. The 15 samples were subtyped by sequencing of the hexon gene; 2 were type 1, 5 were type 2, 7 were type 3, and 1 was type 22. Types 1, 2, and 3 were correctly classified by the GenMark RVP, which differentiates adenovirus positive results into group B/E or group C. The sample sequenced as serotype 22, a group D adenovirus, was called Adeno C.

### Bacterial targets.

The BioFire and Luminex assays both include Mycoplasma pneumoniae in their target menus. Three samples were M. pneumoniae positive by both tests and thus were considered true-positive samples. One additional sample was M. pneumoniae positive by the BioFire RP alone; confirmatory sequencing verified it as a true-positive sample. Previous studies of the BioFire and Luminex assays showed 100% sensitivity for M. pneumoniae in comparison with reference methods, although the number of positive samples was low in each study (7, 13). Other bacterial targets included in the assays used are Bordetella pertussis (BioFire only) and Chlamydia pneumoniae (BioFire and Luminex); however, because none of the samples tested was positive for either of these targets, they were not evaluated in this study.

### Performance of specimens positive for more than one target.

Of the 24 samples that were consensus positive for two targets, 14 were dual positive by all three tests. Nine of the remaining 10 samples had one target that was positive by all three tests and one target for which one of the tests missed a single analyte. There were 3 GenMark false-negative samples (2 with rhinovirus/enterovirus and 1 with RSV), 3 Luminex false-negative samples (1 each with adenovirus, rhinovirus/enterovirus, and PIV-4), and 3 BioFire false-negative samples (1 with adenovirus and 2 with rhinovirus/enterovirus). The remaining discordant dual positive sample was CoV HKU1 positive by GenMark and BioFire and PIV-4 positive by GenMark. There were only 3 triple positive samples, 2 of which were concordant among the three tests. The remaining specimen was positive for adenovirus, PIV-4, and rhinovirus/enterovirus, but the BioFire assay was falsely negative for adenovirus and the Luminex assay was falsely negative for PIV-4.

### Age group-specific performance.

PPA was calculated for each assay in each age group (≤5, 6 to 18, 19 to 64, or ≥65 years of age). Chi-square analysis found no differences between the assays for any age group (*P* value range of 0.831 to 0.997) or between age groups within each assay (*P* value range of 0.922 to 0.955). When the analyses were repeated for NPA, a significant difference was found for patients ≤5 years of age (*P* = 0.0100), driven by the increased number of false-positive results with the GenMark RVP (2 for PIV-4, 2 for rhinovirus, and 1 each for adenovirus, influenza B, MPV, PIV-2, and RSV A), which had a NPA of 55.0% in this age group. The intra-assay NPA for GenMark was not significant, with a *P* value of 0.0581; no other comparisons approached significance. The Luminex RPP was the only assay to achieve 100% NPA in the largest age group, i.e., 19 to 64 years of age.

### Workflow.

The Luminex and GenMark assays are both batched tests that require a separate extraction step and amplification on a thermal cycler. The Luminex RPP is then conveyed directly to the MagPix instrument for detection and analysis, while the GenMark RVP requires a cleanup step, preparation of a hybridization solution, and transfer of the hybridized amplicon to a cartridge for detection and analysis on the XT-8 instrument. Throughput for the Luminex and GenMark assays is limited by the extraction step on the bioMériuex easyMag, which permits up to 24 samples to be processed simultaneously. The MagPix instrument allows a set of up to 96 samples and controls to be analyzed in a single batch, while the capacity of the XT-8 is dependent on its configuration, generally with 8 to 24 concurrent samples. Once the amplicon and hybridization solution are loaded into the GenMark cartridge, it is stable for up to 8 h, permitting staggered runs on the instrument. Time to result for a batch of 24 patient samples and controls is approximately 5.5 h for the Luminex assay and 7.2 h for the GenMark. The BioFire RP incorporates extraction, amplification, detection, melting curve, and analysis within a single system. Each sample requires one cartridge and one FilmArray instrument. Throughput is limited by the number of instruments available, but the time to result for each specimen is ~70 min.

## DISCUSSION

Luminex Molecular Diagnostics brought the first multiplexed respiratory virus panel to market in 2008 and was eventually joined by GenMark Diagnostics and BioFire Diagnostics. In 2015, Luminex launched the NxTAG RPP; in 2017, BioFire launched the RP2 and GenMark the ePlex RP. In this study, we sought to compare the Luminex NxTAG RPP to two common syndromic RPs used in clinical laboratories at the time of this study. When the cumulative data were analyzed, there were no differences in the percent agreements between any of the three assays and the consensus result. Upon closer analysis, however, there are significant differences on a per-analyte basis.

The BioFire RP was the simplest assay to perform and the only sample-to-answer test in this study. The target menu incorporates the same viral panel as the GenMark RVP, with the addition of enterovirus. B. pertussis, C. pneumoniae, and M. pneumoniae are also included, although only M. pneumoniae was evaluated in this study. The overall PPA for the BioFire RP was 94.2%, and the NPA was 95.0%. Six of 7 false-positive results were for *Coronaviridae* (229E, *n* = 1; HKU1, *n* = 1; OC43, *n* = 4); all 4 OC43 false-positive results were in conjunction with HKU1 true-positive results and so may be the product of known cross-reactivity between the two viruses in the BioFire assay (BioFire RP package insert). The only other false-positive result was for MPV. The assay detected 13/15 (86.7%) of the adenovirus-positive samples, 30/31 (96.8%) of the influenza A-positive samples, 14/15 (93.3%) of the influenza B-positive samples, and 32/34 (94.1%) of the RSV-positive samples. BioFire RP was 100% accurate for CoV NL63, PIV-2, PIV-3, and M. pneumoniae. The time from sample processing to result was ~67 min.

The GenMark RVP was the most labor-intensive assay, with an off-board extraction and two postamplification steps, i.e., exonuclease and hybridization-detection. The GenMark RVP offered the fewest targets but the highest overall PPA (97.3%). Of 7 false-negative results, 6 were for rhinovirus/enterovirus and thus could represent enterovirus-positive results, and 1 was for CoV NL63. This is somewhat tempered by the increased number of false-positive results, especially for patients ≤5 years of age. The overall NPA was 81.1%, as a result of false-positive results for influenza B (*n* = 4), rhinovirus (*n* = 10), PIV-1 (*n* = 2), PIV-2 (*n* = 3), PIV-4 (*n* = 4), and 1 each of adenovirus, MPV, OC43, and RSV. The GenMark assay was 100% accurate for CoV 229E, CoV HKU1, influenza A, and PIV-3. For a batch of 21 samples and associated controls, the time from sample processing to result was ~7.2 h.

The Luminex RPP assay had the broadest selection of targets with 20, including bocavirus and two bacterial targets (C. pneumoniae and M. pneumoniae). The relative ease of use was improved, compared to the initial Luminex xTAG RVP assay, which required separate extraction, amplification, exonuclease, secondary amplification, and hybridization steps. The Luminex NxTAG RPP assay still involves off-board extraction, but the eluate is added directly to lyophilized amplification and detection reagents, eliminating the need for making master mix or hybridization solution. Postamplification, the tray is transferred to the MagPix instrument for detection without additional manipulation. The RPP was the only one of the three tests evaluated with the capacity to detect human bocavirus. Fourteen NP swab samples tested positive for bocavirus; 11 were from patients 2 years of age or younger. All were submitted for confirmatory testing by bidirectional sequencing; 8 were confirmed as true-positive samples, 1 gave an indeterminate result, and 5 were negative.

The Luminex RPP detected 100% of CoV 229E, NL63, and OC43, as well as MPV, influenza A and B, PIV-1 to PIV-3, and RSV. The overall PPA was 96.3%, with 93.8% NPA. For a batch of 21 samples and associated controls, the time from sample processing to result was ~2.75 h.

Because this was a retrospective study with samples selected to challenge the assays, we were unable to calculate sensitivity and specificity or to determine predictive values. Also, individual reference tests were unavailable for every assay, necessitating the use of a consensus standard of 2 of 3 assays. These approaches can lead to biased results, because a more sensitive assay might appear to have increased numbers of false-positive results. Another potential source of bias is that the samples were tested with the GenMark RVP assay clinically, and those results were used in selecting specimens to be used for the study. Finally, an additional freeze/thaw cycle occurred prior to testing on the BioFire platform due to the need to ship specimens for testing by FilmArray. Since the time of this study, BioFire and GenMark have developed new products, which were not part of this evaluation. The Luminex NxTAG RPP assay used in this study is currently available in addition to an Emergency Use Authorization (EUA) version with SARS-CoV-2. The inclusion of SARS-CoV-2 in RPs is now essential, and it has also been added to the GenMark and BioFire RPs.

Outcome studies have shown that multiplex respiratory pathogen testing can have positive impacts on patient care by decreasing the amount or duration of prescribed antibiotics, increasing the use of oseltamivir for influenza A-positive patients, reducing the time spent in isolation, and even reducing the overall lengths of stay for hospitalized children (17, 18). In adults, rapid, accurate diagnosis of respiratory infections has been shown to lower odds ratios for admission, length of stay, and duration of antimicrobial use (19).

There are now numerous FDA-cleared multiplex assays available for respiratory pathogen detection. We chose to focus on the three broadest panels. All three had high PPA and NPA values. The core menus of each panel are the same, with the exception of the lack of enterovirus and bacterial pathogens for the GenMark RVP. While the BioFire RP has the easiest workflow, the Luminex NxTAG RPP provides a compressed workflow with high throughput and similar performance characteristics, compared to BioFire RP and GenMark eSensor RVP.

## MATERIALS AND METHODS

### Study specimens.

The NP swab specimens (*n* = 350) from 329 symptomatic patients were used in this study; 19 patients had 2 specimens included and 1 patient had 3. For patients with more than 1 swab sample included, the average interval between swab collection dates was 47 days. Convenience sampling was used to enrich for positive specimens for each panel target. Patients fell into the following age groups: ≤5 years (*n* = 69), 6 to 18 years (*n* = 26), 19 to 64 years (*n* = 160), and ≥65 years (*n* = 74). Specimens were collected between January 2015 and July 2016, predominately between November 2015 and April 2016 (*n* = 325). Flocked swab samples were preserved in universal viral transport medium (BD, Sparks, MD) and stored at −70°C until the time of study testing. Preservation of cellular nucleic acid during storage of eluted flocked swab samples has been shown previously (20).

### Respiratory virus testing.

All NP swab samples were tested with the BioFire FilmArray RP, GenMark eSensor RVP, and Luminex NxTAG RPP assays according to their respective package inserts. Targets included for each test are shown in Table 3. Fresh specimens were tested at the time of collection with the GenMark eSensor RVP and reported in the patient chart according to routine laboratory protocol. Testing by BioFire RP and Luminex NxTAG RPP was batched and performed on thawed frozen specimens in 2016. Nucleic acid extraction for the GenMark and Luminex tests was performed using the EasyMag system (bioMérieux, Durham, NC) and was completed within 72 h after collection (GenMark) or within 24 h after thawing of the sample (Luminex). Aliquots were created and refrozen at the time of thawing for future shipments to Memorial Sloan Kettering Cancer Center, where the BioFire testing was conducted. Shipments were batched and sent overnight on dry ice. All samples were also tested using our adenovirus-specific LDT; extraction for this test was performed using the MagNA Pure system (Roche, Indianapolis, IN). The LDT utilized the ABI 7500 real-time PCR system with primers and probes described previously (21). Adenovirus typing was performed by sequencing the hexon gene according to the protocol of Lu and Erdman (22).

**TABLE 3:** Targets detected by each test

Target	Detection by:		
	BioFire FilmArray RP	GenMark eSensor RVP	Luminex NxTAG RPP
Adenovirus	X	B/E, C	X
CoVs 229E, HKU1, NL63, and OC43	X	X^*a*^	X
Bocavirus			X
MPV	X	X	X
Rhinovirus/enterovirus	X	Rhinovirus only	X
Influenza A	H1/H3/H1-2009	H1/H3/H1-2009	H1/H3/H1-2009
Influenza B	X	X	X
PIV-1 to PIV-3	X	X	X
PIV-4	X	X^*a*^	X
RSV	X	A/B	A/B
Bordetella pertussis	X		
Chlamydia pneumoniae	X		X
Mycoplasma pneumoniae	X		X

a Detected when analyzed in RUO mode.

### Reference results.

The interpretation of results was performed according to the individual package inserts. The GenMark RVP was analyzed using the Research Use Only (RUO) software to include the *Coronaviridae* and PIV-4. Specimens with indeterminate or equivocal results were repeated according to the manufacturers’ suggestions. A true-positive (i.e., consensus-positive) result was defined as being positive by two or more of the platforms or positive by the adenovirus LDT. All samples that were positive for bocavirus by Luminex and 1 sample with an unconfirmed M. pneumoniae result were analyzed with bidirectional sequencing using analytically validated primers that were directed against different genomic regions, compared with the Luminex assay primers. Where sequencing results were available, they were considered true results.

### Statistical analysis.

All analyses were performed using GraphPad software (GraphPad, La Jolla, CA). *P* values of ≤0.05 were considered significant.

This study was approved by the University of North Carolina at Chapel Hill Institutional Review Board.

## References

[1] Schuchat A, Anderson LJ, Rodewald LE, Cox NJ, Hajjeh R, Pallansch MA, Messonnier NE, Jernigan DB, Wharton M. 2018. Progress in vaccine-preventable and respiratory infectious diseases: first 10 years of the CDC National Center for Immunization and Respiratory Diseases, 2006–2015. Emerg Infect Dis 24:1178–1187. doi:10.3201/eid2407.171699. 29916350 PMC6038744

[2] Jain S, Self WH, Wunderink RG, Fakhran S, Balk R, Bramley AM, Reed C, Grijalva CG, Anderson EJ, Courtney DM, Chappell JD, Qi C, Hart EM, Carroll F, Trabue C, Donnelly HK, Williams DJ, Zhu Y, Arnold SR, Ampofo K, Waterer GW, Levine M, Lindstrom S, Winchell JM, Katz JM, Erdman D, Schneider E, Hicks LA, McCullers JA, Pavia AT, Edwards KM, Finelli L, CDC EPIC Study Team. 2015. Community-acquired pneumonia requiring hospitalization among U.S. adults. N Engl J Med 373:415–427. doi:10.1056/NEJMoa1500245. 26172429 PMC4728150

[3] Jain S, Williams DJ, Arnold SR, Ampofo K, Bramley AM, Reed C, Stockmann C, Anderson EJ, Grijalva CG, Self WH, Zhu Y, Patel A, Hymas W, Chappell JD, Kaufman RA, Kan JH, Dansie D, Lenny N, Hillyard DR, Haynes LM, Levine M, Lindstrom S, Winchell JM, Katz JM, Erdman D, Schneider E, Hicks LA, Wunderink RG, Edwards KM, Pavia AT, McCullers JA, Finelli L. 2015. Community-acquired pneumonia requiring hospitalization among U.S. children. N Engl J Med 372:835–845. doi:10.1056/NEJMoa1405870. 25714161 PMC4697461

[4] Fleming-Dutra KE, Hersh AL, Shapiro DJ, Bartoces M, Enns EA, File TM, Jr, Finkelstein JA, Gerber JS, Hyun DY, Linder JA, Lynfield R, Margolis DJ, May LS, Merenstein D, Metlay JP, Newland JG, Piccirillo JF, Roberts RM, Sanchez GV, Suda KJ, Thomas A, Woo TM, Zetts RM, Hicks LA. 2016. Prevalence of inappropriate antibiotic prescriptions among US ambulatory care visits, 2010–2011. JAMA 315:1864–1873. doi:10.1001/jama.2016.4151. 27139059

[5] Harada Y, Kinoshita F, Yoshida LM, Minh LN, Suzuki M, Morimoto K, Toku Y, Tomimasu K, Moriuchi H, Ariyoshi K. 2013. Does respiratory virus coinfection increases the clinical severity of acute respiratory infection among children infected with respiratory syncytial virus? Pediatr Infect Dis J 32:441–445. doi:10.1097/INF.0b013e31828ba08c. 23838658

[6] World Health Organization. 2014. Infection prevention and control of epidemic- and pandemic-prone acute respiratory infections in health care. World Health Organization, Geneva, Switzerland. https://www.who.int/publications/i/item/infection-prevention-and-control-of-epidemic-and-pandemic-prone-acute-respiratory-infections-in-health-care. 24983124

[7] Tang YW, Gonsalves S, Sun JY, Stiles J, Gilhuley KA, Mikhlina A, Dunbar SA, Babady NE, Zhang H. 2016. Clinical evaluation of the Luminex NxTAG Respiratory Pathogen Panel. J Clin Microbiol 54:1912–1914. doi:10.1128/JCM.00482-16. 27122378 PMC4922127

[8] Popowitch EB, O'Neill SS, Miller MB. 2013. Comparison of the Biofire FilmArray RP, Genmark eSensor RVP, Luminex xTAG RVPv1, and Luminex xTAG RVP fast multiplex assays for detection of respiratory viruses. J Clin Microbiol 51:1528–1533. doi:10.1128/JCM.03368-12. 23486707 PMC3647947

[9] Gonsalves S, Mahony J, Rao A, Dunbar S, Juretschko S. 2019. Multiplexed detection and identification of respiratory pathogens using the NxTAG® Respiratory Pathogen Panel. Methods 158:61–68. doi:10.1016/j.ymeth.2019.01.005. 30660863 PMC7128802

[10] Chan KH, To KKW, Li PTW, Wong TL, Zhang R, Chik KKH, Chan G, Yip CCY, Chen HL, Hung IFN, Chan JFW, Yuen KY. 2017. Evaluation of NxTAG Respiratory Pathogen Panel and comparison with xTAG Respiratory Viral Panel Fast v2 and Film Array Respiratory Panel for detecting respiratory pathogens in nasopharyngeal aspirates and swine/avian-origin influenza A subtypes in culture isolates. Adv Virol 2017:1324276. doi:10.1155/2017/1324276. 28947901 PMC5602486

[11] Lee CK, Lee HK, Ng CW, Chiu L, Tang JW, Loh TP, Koay ES. 2017. Comparison of Luminex NxTAG Respiratory Pathogen Panel and xTAG Respiratory Viral Panel FAST version 2 for the detection of respiratory viruses. Ann Lab Med 37:267–271. doi:10.3343/alm.2017.37.3.267. 28224774 PMC5339100

[12] Esposito S, Scala A, Bianchini S, Presicce ML, Mori A, Sciarrabba CS, Fior G, Principi N. 2016. Partial comparison of the NxTAG Respiratory Pathogen Panel assay with the Luminex xTAG Respiratory Panel Fast Assay V2 and singleplex real-time polymerase chain reaction for detection of respiratory pathogens. Diagn Microbiol Infect Dis 86:53–57. doi:10.1016/j.diagmicrobio.2016.06.018. 27401400 PMC7132749

[13] Chen JHK, Lam HY, Yip CCY, Wong SCY, Chan JFW, Ma ESK, Cheng VCC, Tang BSF, Yuen KY. 2016. Clinical evaluation of the new high-throughput Luminex NxTAG Respiratory Pathogen Panel assay for multiplex respiratory pathogen detection. J Clin Microbiol 54:1820–1825. doi:10.1128/JCM.00517-16. 27122380 PMC4922091

[14] Beckmann C, Hirsch HH. 2016. Comparing Luminex NxTAG-Respiratory Pathogen Panel and RespiFinder-22 for multiplex detection of respiratory pathogens. J Med Virol 88:1319–1324. doi:10.1002/jmv.24492. 26856438 PMC7166946

[15] Brotons P, Henares D, Latorre I, Cepillo A, Launes C, Munoz-Almagro C. 2016. Comparison of NxTAG Respiratory Pathogen Panel and Anyplex II RV16 tests for multiplex detection of respiratory pathogens in hospitalized children. J Clin Microbiol 54:2900–2904. doi:10.1128/JCM.01243-16. 27629904 PMC5121377

[16] Sails AD, Eltringham G, Valappil M, Waugh S, Saunders D. 2017. Comparison of the Luminex NxTAG respiratory pathogen panel and a multiplex in-house real-time PCR panel for the detection of respiratory viruses in symptomatic patients. J Med Microbiol 66:1291–1296. doi:10.1099/jmm.0.000562. 28868996

[17] Lee BR, Hassan F, Jackson MA, Selvarangan R. 2019. Impact of multiplex molecular assay turn-around-time on antibiotic utilization and clinical management of hospitalized children with acute respiratory tract infections. J Clin Virol 110:11–16. doi:10.1016/j.jcv.2018.11.006. 30502640 PMC7106386

[18] Rogers BB, Shankar P, Jerris RC, Kotzbauer D, Anderson EJ, Watson JR, O'Brien LA, Uwindatwa F, McNamara K, Bost JE. 2015. Impact of a rapid respiratory panel test on patient outcomes. Arch Pathol Lab Med 139:636–641. doi:10.5858/arpa.2014-0257-OA. 25152311

[19] Rappo U, Schuetz AN, Jenkins SG, Calfee DP, Walsh TJ, Wells MT, Hollenberg JP, Glesby MJ. 2016. Impact of early detection of respiratory viruses by multiplex PCR assay on clinical outcomes in adult patients. J Clin Microbiol 54:2096–2103. doi:10.1128/JCM.00549-16. 27225406 PMC4963510

[20] Castriciano S, Petrich A, Smieja M, Chernesky M. 2008. Diagnostic outcomes of viral samples collected by flocked swabs, stored in UTM at −70C and tested by culture, direct fluorescence or molecular assays. Abstr Eur Soc Clin Virol Annu Meet, Saariselkä, Lapland, Finland.

[21] Heim A, Ebnet C, Harste G, Pring-Akerblom P. 2003. Rapid and quantitative detection of human adenovirus DNA by real-time PCR. J Med Virol 70:228–239. doi:10.1002/jmv.10382. 12696109

[22] Lu X, Erdman DD. 2006. Molecular typing of human adenoviruses by PCR and sequencing of a partial region of the hexon gene. Arch Virol 151:1587–1602. doi:10.1007/s00705-005-0722-7. 16502282

