# Supplementary material for: Comparative Performance of the Luminex NxTAG Respiratory Pathogen Panel, GenMark eSensor Respiratory Viral Panel, and BioFire FilmArray Respiratory Panel
Source: Microbiol Spectr. 2022 Jun 29;10(4):e01248-22. doi: 10.1128/spectrum.01248-22 (PMC9431521; doi:10.1128/spectrum.01248-22)
Supplement: Supplemental file 1 — Table S1. Download spectrum.01248-22-s0001.pdf, PDF file, 0.1 MB [file spectrum.01248-22-s0001.pdf]

Supplemental Table 1. Analysis and comparison of the accuracy of the tests by target analyte. The numbers of true positives (T.P.), false positives (F.P.) and false negatives (F.N.) are shown, along with the positive percent agreement (PPA). McNemar's test (with continuity correction) was used to compare each assay to the others. Statistically significant differences are noted with an asterisk.

Adenovirus					Influenza A				
	T.P.	F.P.	F.N.	*PPA*		T.P.	F.P.	F.N.	*PPA*
BF RP	13	0	2	*86.7*	BF RP	30	0	1	*96.8*
GM RVP	15	1	0	*100*	GM RVP	31	0	0	*100*
LX RPP	14	0	1	*93.3*	LX RPP	31	1	0	*100*

Adenovirus: BF/GM p=0.2482; BF/LX p=1; GM/LX p=0.4795

Influenza A: BF/GM p=1; BF/LX p=0.4795; GM/LX p=1

Coronaviridae					Influenza B				
	T.P.	F.P.	F.N.	*PPA*		T.P.	F.P.	F.N.	*PPA*
BF RP	42	6	0	*100*	BF RP	14	0	1	*93.3*
GM RVP	41	1	1	*97.6*	GM RVP	15	4	0	*100*
LX RPP	40	0	2	*95.2*	LX RPP	15	0	0	*100*

Coronaviridae: BF/GM p=0.0771; *BF/LX p=0.0412; GM/LX p=0.4795

Influenza B: BF/GM p=0.0736; BF/LX p=1; GM/LX p=0.1336

Metapneumovirus					Parainfluenza viruses 1 - 4				
	T.P.	F.P.	F.N.	*PPA*		T.P.	F.P.	F.N.	*PPA*
BF RP	22	1	0	*100*	BF RP	28	0	3	*90.3*
GM RVP	22	1	0	*100*	GM RVP	31	9	0	*100*
LX RPP	22	2	0	*100*	LX RPP	29	0	2	*93.6*

Metapneumovirus: BF/GM p=0.4795; BF/LX p=1; GM/LX p=1

Parainfluenza viruses 1 - 4: *BF/GM p=0.0015; BF/LX p=1; GM/LX p=0.0704

Rhinovirus/Enterovirus					Respiratory Syncytial Virus				
	T.P.	F.P.	F.N.	*PPA*		T.P.	F.P.	F.N.	*PPA*
BF RP	60	0	6	*90.9*	BF RP	32	0	2	*94.1*
GM RVP	60	10	6	*90.9*	GM RVP	34	1	0	*100*
LX RPP	62	1	4	*93.9*	LX RPP	34	1	0	*100*

Rhinovirus/Enterovirus: BF/GM p=0.0550; BF/LX p=0.5465; GM/LX p=0.1904

Respiratory Syncytial Virus: BF/GM p=0.2482; BF/LX p=0.2482; GM/LX p=0.4795
